# Threats of COVID-19 to Achieving United Nations Sustainable Development Goals in Africa

**DOI:** 10.4269/ajtmh.20-1489

**Published:** 2020-12-15

**Authors:** Osmond C. Ekwebelem, Ekenedirichukwu S. Ofielu, Obinna V. Nnorom-Dike, Chizoba Iweha, Nicholas C. Ekwebelem, Bright C. Obi, Sani E. Ugbede-Ojo

**Affiliations:** 1 Research and Development Hub, University of Nigeria, Nsukka, Nigeria;; 2 Faculty of Biological Sciences, University of Nigeria, Nsukka, Nigeria;; 3 Department of Economics and Statistics, University of Benin, Benin, Nigeria;; 4 Faculty of Clinical Sciences, University of Port-Harcourt, Port-Harcourt, Nigeria;; 5 Faculty of Law, Kogi State Universtiy, Anyigba, Nigeria

## Abstract

The negative impacts of the COVID-19 pandemic have been exacerbated in Africa by hunger, poor health care, poor educational systems, poverty, and lack of potable water and sanitation. With the pandemic and a worrying global recession as a result of COVID-19, our ability to achieve the 17 United Nations sustainable development goals (SDGs) in the post-pandemic era has been questioned. There is concern that the economic stagnation caused by COVID-19 will not only push more populations below the poverty line but also limit international support to ensure progress toward achieving the SDGs in Africa. This article highlights how the COVID-19 pandemic could threaten the actualization of the SDGs in Africa. We assessed relevant published literature, observations, and current global trends. Our results suggest that although the improvement of healthcare systems has become a priority in Africa, there is a need to ensure that some SDGs are not sacrificed to achieve control of the pandemic. Despite the pandemic, African countries need to identify policies that will not compromise the implementation of the SDGs and/or jeopardize previously achieved SDG targets.

## INTRODUCTION

COVID-19 was officially declared a public health emergency of international concern by the WHO on January 30, 2020.^1^ The disease caused by the COVID-19 has since affected almost every corner of the world, with about 216 countries and territories affected. The countries in Africa with the highest number of confirmed COVID-19 cases are South Africa, Morocco, Egypt, Ethiopia, and Nigeria.^2^ The current pandemic has resulted in a health crisis that has seriously impacted human lives and the world economy.^3^ Unfortunately, the amount of time required to curtail the spread of the virus and return back to normalcy is uncertain. The sustainable development goals (SDGs) are a group of 17 interconnected goals with 169 targets designed to serve as a road map to achieve a better and more sustainable future for all by 2030. At this unprecedented time, SDG 3—good health and well-being—has justifiably been on the top of the priority list of all governments, thus raising the concern that some SDGs are being neglected or sacrificed to achieve control of the pandemic. According to global health experts, the current health crisis will be given the highest priority until a lasting solution in the form of a readily available vaccine becomes available.^4,5^

The SDGs were inaugurated in 2015 by the United Nations (UN), but almost all countries in Africa are lagging behind in the race to achieve the goals by 2030.^6^ Economic stagnation in Africa, coupled with the current global recession caused by COVID-19, is predicted to lead to COVID-19’s impacts being more vicious and long term than those of the 2008–2009 global economic crisis.^7^ Although the UN is optimistic with its plan to “defeat the virus and build a better world,”^8^ these negative impacts will seriously threaten the actualization of the UN SDGs, in most African countries. Thus, the UN strategic plan calls for global solidarity and innovative, inclusive, decisive, and coordinated actions by industrialized countries, which entails technical and financial support for poor and vulnerable regions.^8^ As a way forward, the UN approved an appeal to raise 2 billion United States dollar (USD) to help in the battle against COVID-19.^9^ This move has been described as absolutely necessary as the current pandemic may lead to a “famine of biblical proportions.”^10^ This study examines how the pandemic will threaten the implementation of the SDGs in Africa. It also highlights the SDGs most directly threatened by COVID-19 and for which areas urgent attention is most needed.

## SOCIOECONOMIC IMPACT OF COVID-19 IN AFRICA

The main socioeconomic impacts of COVID-19 on African countries are still unclear,^11^ and the literature evaluating them is still limited. Few studies that have evaluated the socioeconomic impacts of COVID-19 in Africa have focused on specific sectors such as health care,^12^ tourism,^13^ mining,^14^ or the economy.^3,15^ It is estimated that the economic stagnation caused by COVID-19 will push 420–580 million people into poverty, consequently elevating global poverty for the first time since 1990.^16^ In view of this, global leaders are encouraged to commit to an economic recovery initiative that will support high-risk economies and their vulnerable communities.^17^ A recent article,^18^ which focused on the socioeconomic impact of COVID-19 on four African countries (Ethiopia, Malawi, Nigeria, and Uganda), reported that an estimated 258 million people, making up 80% of the population of the four countries, have lost their source of livelihood because of COVID-19. Coupled with the food insecurity that existed even before the pandemic, it is predictable that this pandemic will push more populations below the poverty line, a phenomenon perceived as a threat to the targets of SDG 1, SDG 2, SDG 8, and SDG 10 (Table 1).

**Table 1:** Threatened Sustainable Development Goals in Africa

SDG	Status	Target(s) threatened
SDG 1: No poverty	Threatened	Target 1.2: halve proportion of people living in poverty by 2030
		Target 1.4: provide equal access to basic service
SDG 2: Zero hunger	Threatened	Target 2.3: double agricultural productivity and incomes of small-scale food producers
SDG 3: Good health and well-being	Threatened	Target 3.8: achieve universal health coverage
SDG 4: Quality education	Threatened	Target 4.1: provide free, equitable, and quality education for all children
SDG 6: Clean water and sanitation	Threatened	Target 6.1: give access to safe and affordable drinking water for all
SDG 8: Decent work and economic growth	Threatened	Target 8.1: sustain per capita economic growth
SDG 10: Reduced inequalities	Threatened	Target 10.1: sustain above-average income growth of the bottom 40% of the population
SDG 16: Peace, justice, and strong institutions	Threatened	Target 16.1: reduce all forms of violence and related deaths everywhere
SDG 17: Partnerships for the goals	Threatened	Target 17.2: developed countries should commit at least 0.7% of gross national income in overseas aid for developing and 0.2% to least-developed nations

SDG = sustainable development goal.

The financial market in Africa was badly hit by the COVID-19 pandemic. Countries such as South Africa, Morocco, and Kenya recorded declines in all share indexes after the first coronavirus case was announced in each country.^11^ South Africa and Kenya witnessed 80% and 55% fall, respectively, in tourism following the onset of COVID-19.^19,20^ A significant drop in oil price heavily impacted some oil export–dependent countries including Nigeria and Angola.^15^ Because the estimated oil price of USD 57 per barrel was no longer sustainable because of COVID-19, Nigeria revised its budgets downward to USD 30 per barrel. Countries such as Zambia and Angola are considering debt restructuring because the debt-to-gross domestic product ratio, which was about 70% in these countries in 2018, has increased.^11^ The pandemic also took a sociological toll on vulnerable populations in Africa, particularly the elderly, people in impoverished homes, and persons with disabilities. Refugees, migrants, and internally displaced persons suffered disproportionately because of lack of running water and limited employment opportunities.^21^ It is forecasted that failure to address these problems through affordable social policy will amplify social exclusion, inequality, and unemployment in Africa.^3,15,22^ These problems are a clear threat to achieving the existing SDG targets (Table 1).

## THREAT OF COVID-19 TO ACHIEVING SDGS IN AFRICA

The UN SDGs were established based on the three main concepts of sustainability, which are economic, social, and environmental.^23^ However, unlike the Millennium Development Goals, which were focused on battling poverty in developing countries over 15 years (2000–2015), the SDGs are considered universally applicable to all societies around the world. The commitment to implement the SDGs within 15 years (2015–2030) has been unanimously accepted by all nations. Before COVID-19, African countries were considered to be lagging behind in the quest to achieve the SDG targets, and clearly, this pandemic will endanger the little progress made so far. Highly threatened SDGs and SDG target(s) in Africa are listed in Table 1.

It is increasingly likely that all the 169 SDG targets will not be accomplished within the targeted 15 years (2015–2030). Analysts believe that two-thirds of the 169 targets are under threat because of the COVID-19 pandemic.^4,23,24^ Threats on SDG 1 (no poverty) and SDG 2 (zero hunger) are not the only perceived threats from COVID-19 to the social aspects of sustainability in Africa. Lessons from our past pandemics shine a spotlight on the significant impact a pandemic can leave psychologically,^25^ without doubt exacerbated by future uncertainty and socioeconomic inequalities in Africa. Hundreds of millions of people, especially in developing and least-developed nations, have no health insurance, no unemployment insurance, and no income security.^26^ Undoubtedly, these poor living conditions will be amplified by the pandemic, a situation seen as a major blow to the main goals of sustainable development, which are inclusiveness and leaving no one behind.^27^

Sustainable development goal 4 (quality education), SDG 6 (clean water and sanitation), SDG 8 (decent work and economic growth), and SDG 10 (reduced inequality) are unequivocally threatened by COVID-19 because of diversion of funds and priorities accorded to SDG 3. Such diversion of funds and priority was demonstrated on March 3, 2020, when the World Bank apportioned 12 billion USD emergency funding to help developing and least developed countries in South Asia and sub-Saharan Africa to reinforce their healthcare systems against the pandemic.^28^ The shocking lack of resilience in this system,^29,30^ due to underinvestment in healthcare systems in Africa, is now widely acknowledged. Nonetheless, further addressing these healthcare shortcomings may directly and indirectly endanger the global investments needed to implement and actualize other SDGs. Meanwhile, industrialized countries can be expected to prioritize investments into their own economic recoveries and national emergency programs,^31,32^ threatening a target of SDG 17, which directs developed countries to commit at least 0.7% of gross national income as aid for developing and 0.2% as aid for least-developed nations. Moving forward, global solidarity and shared responsibility will be instrumental in ensuring we recover from losses and regain momentum toward achieving the SDGs in the post-pandemic era.^33^

## FUTURE PERSPECTIVES

Globalization and progressive economies are perceived as the backbone that will help guarantee the success of the SDGs^23^; sadly, these assumptions are now in shambles owing to the current pandemic. The global economy, expected to grow by at least 5% in 2020, is currently in recession,^16^ and developed nations are reluctant to extend financial support to less industrialized countries because they are also struggling to support their own citizens. The world is predicted to face more challenges in the next decade. These challenges range from more pandemics to continued ecosystem degradation, wildfires, floods, storms, and drought, which are considered as consequences of climate change.^23^ In Africa, a weak healthcare sector continues to struggle with malaria, AIDS, tuberculosis, dengue, and other tropical diseases. These looming challenges will further impact the SDGs because these global stressors will lead to the diversion of more funds from their realization. Therefore, current circumstances urge us to reconsider the resilience of the SDGs in the face of such global stressors.

Economic progress and the social well-being of millions of people in Africa have been badly hit by the COVID-19 pandemic, and without a solution in the form of a readily available vaccine, economic activities and implementation of SDGs will continue to be held back. Therefore, African nations should bring into play the lessons learned from this unprecedented crisis and use these lessons as guides for building a more resilient society, capable of withstanding future global stressors that may hinder the realization of the SDGs. It should be acknowledged that the efforts of charitable and philanthropic organizations have played a huge role in combating the difficult times brought on by the pandemic. Moving forward, we need to replicate these efforts in our commitment to achieve the existing SDG targets in Africa during the post–COVID-19 era.

With 10 years remaining in the 15-year life span of the SDGs, their further implementation must not be delayed, despite recent socioeconomic stresses. Governments in Africa should develop policies that are affordable and capable of achieving SDG targets.^21^ Furthermore, as we continue to emphasize the need to implement policies to achieve the SDGs, it is also imperative to understand the power of shared responsibility, which involves extending responsibility from governments to the global community, the private sector, humanitarian and philanthropic organizations, and civil society.^33^ Although the road ahead may seem uncertain, we envision that a few years from now, the catastrophe caused by this pandemic will be remembered as a positive turning point, after which we showed resilience and came back stronger.
